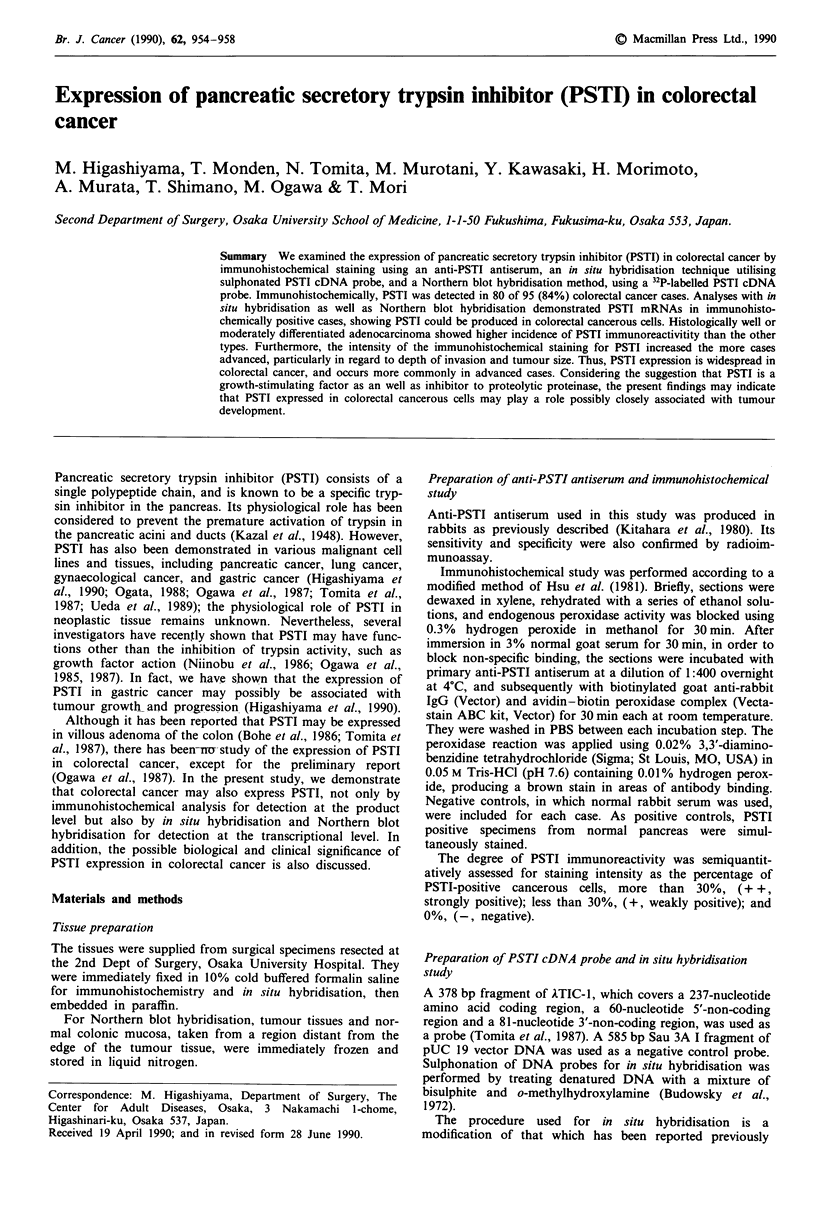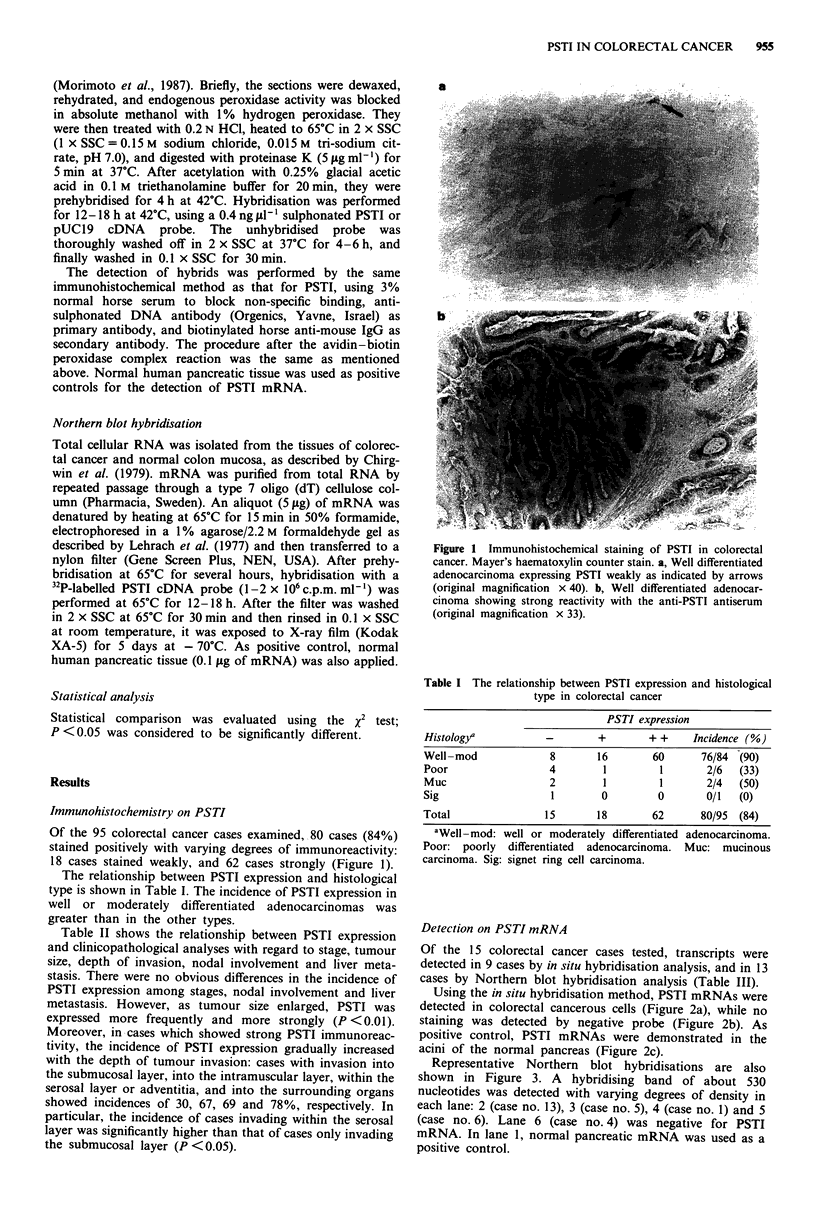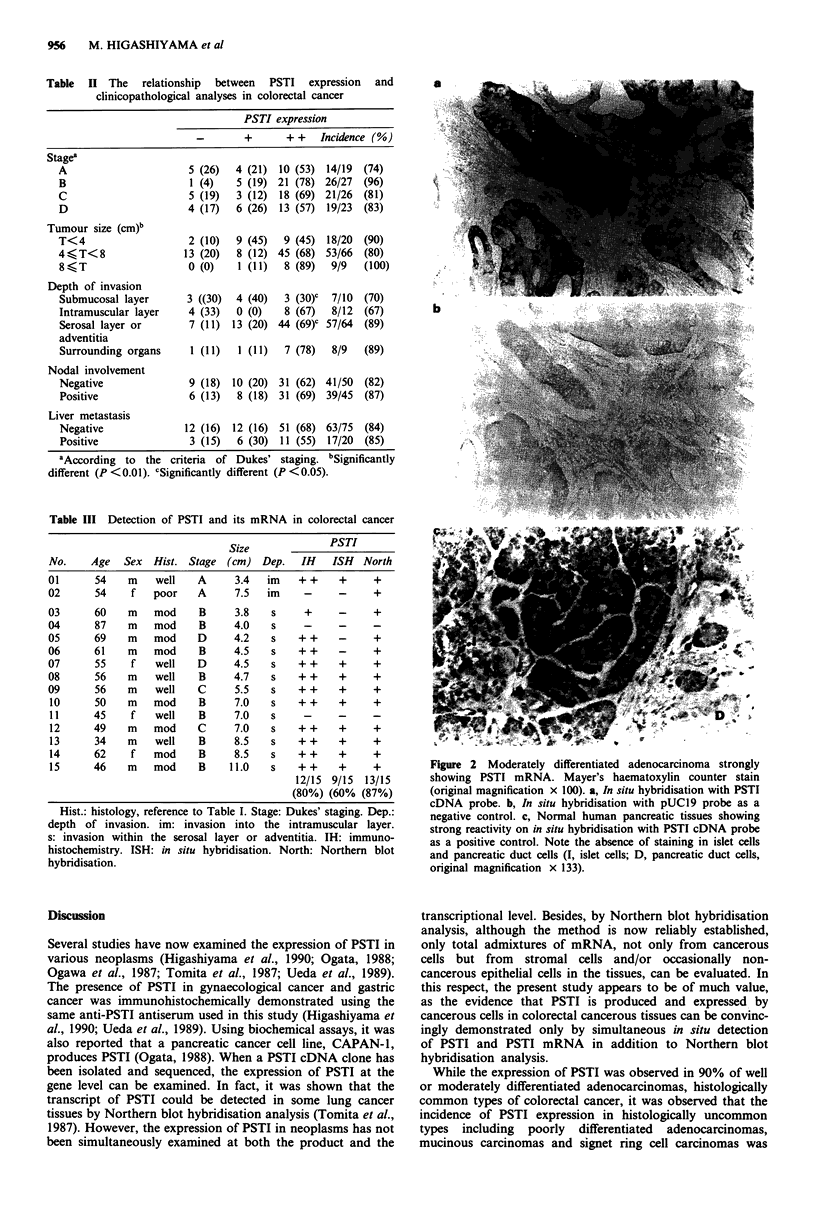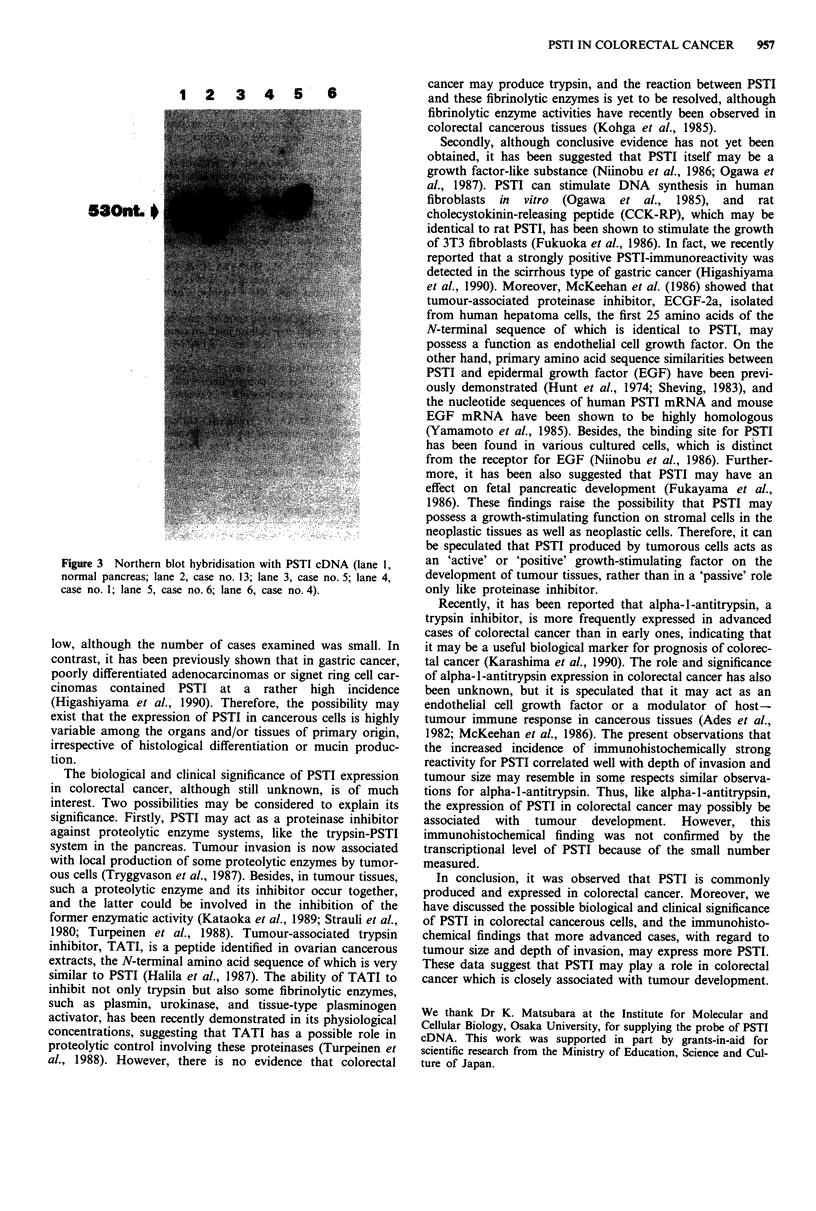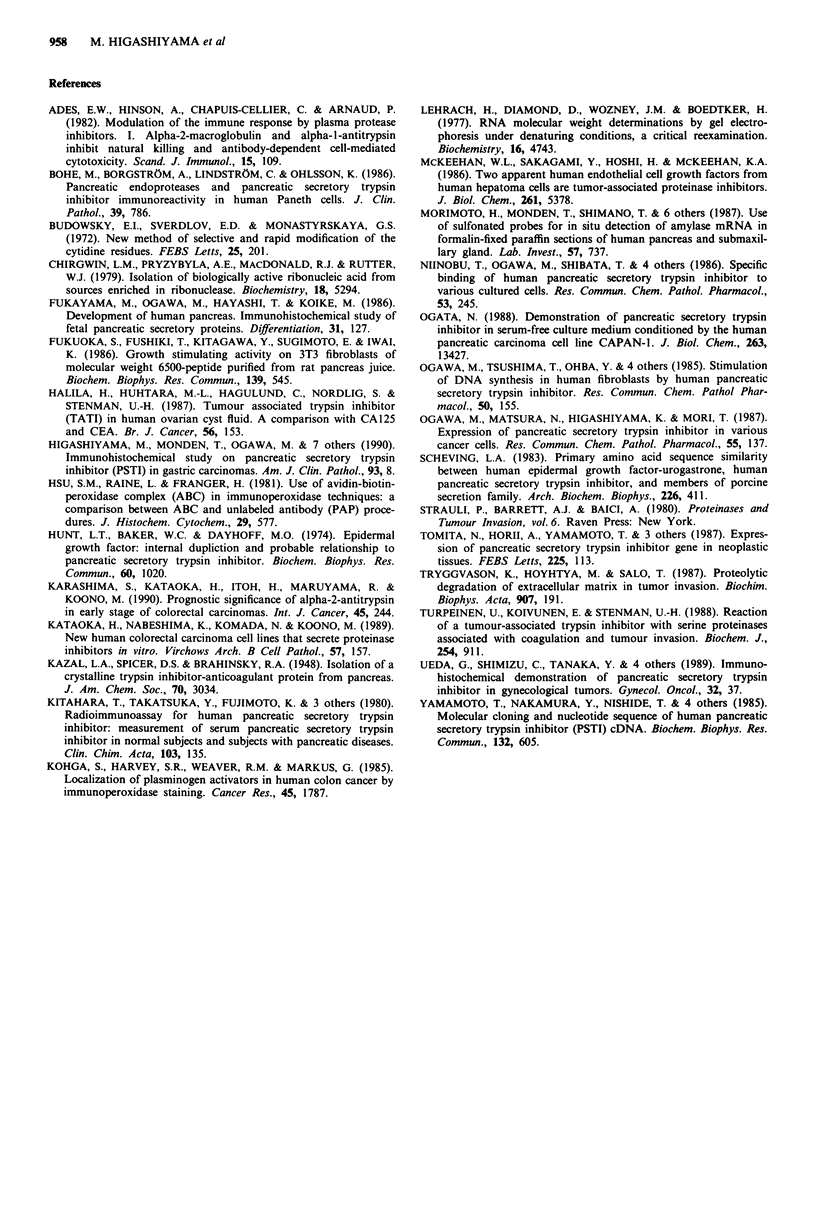# Expression of pancreatic secretory trypsin inhibitor (PSTI) in colorectal cancer.

**DOI:** 10.1038/bjc.1990.416

**Published:** 1990-12

**Authors:** M. Higashiyama, T. Monden, N. Tomita, M. Murotani, Y. Kawasaki, H. Morimoto, A. Murata, T. Shimano, M. Ogawa, T. Mori

**Affiliations:** Second Department of Surgery, Osaka University School of Medicine, Japan.

## Abstract

**Images:**


Br. J. Cancer (1990), 62, 954–958 © Macmillan Press Ltd., 1990

### Expression of pancreatic secretory trypsin inhibitor (PSTI) in colorectal cancer

M. Higashiyama, T. Monden, N. Tomita, M. Murotani, Y. Kawasaki, H. Morimoto, A. Murata, T. Shimano, M. Ogawa & T. Mori

*Second Department of Surgery, Osaka University School of Medicine, 1-1-50 Fukushima, Fukusima-ku, Osaka 553, Japan.*

**Summary** We examined the expression of pancreatic secretory trypsin inhibitor (PSTI) in colorectal cancer by immunohistochemical staining using an anti-PSTI antiserum, an *in situ* hybridisation technique utilising sulphonated PSTI cDNA probe, and a Northern blot hybridisation method, using a $^{32}P$-labelled PSTI cDNA probe. Immunohistochemically, PSTI was detected in 80 of 95 (84%) colorectal cancer cases. Analyses with *in situ* hybridisation as well as Northern blot hybridisation demonstrated PSTI mRNAs in immunohistochemically positive cases, showing PSTI could be produced in colorectal cancerous cells. Histologically well or moderately differentiated adenocarcinoma showed higher incidence of PSTI immunoreactivitity than the other types. Furthermore, the intensity of the immunohistochemical staining for PSTI increased the more cases advanced, particularly in regard to depth of invasion and tumour size. Thus, PSTI expression is widespread in colorectal cancer, and occurs more commonly in advanced cases. Considering the suggestion that PSTI is a growth-stimulating factor as an well as inhibitor to proteolytic proteinase, the present findings may indicate that PSTI expressed in colorectal cancerous cells may play a role possibly closely associated with tumour development.

Pancreatic secretory trypsin inhibitor (PSTI) consists of a single polypeptide chain, and is known to be a specific trypsin inhibitor in the pancreas. Its physiological role has been considered to prevent the premature activation of trypsin in the pancreatic acini and ducts (Kazal *et al.*, 1948). However, PSTI has also been demonstrated in various malignant cell lines and tissues, including pancreatic cancer, lung cancer, gynaecological cancer, and gastric cancer (Higashiyama *et al.*, 1990; Ogata, 1988; Ogawa *et al.*, 1987; Tomita *et al.*, 1987; Ueda *et al.*, 1989); the physiological role of PSTI in neoplastic tissue remains unknown. Nevertheless, several investigators have recently shown that PSTI may have functions other than the inhibition of trypsin activity, such as growth factor action (Niinobu *et al.*, 1986; Ogawa *et al.*, 1985, 1987). In fact, we have shown that the expression of PSTI in gastric cancer may possibly be associated with tumour growth and progression (Higashiyama *et al.*, 1990).

Although it has been reported that PSTI may be expressed in villous adenoma of the colon (Bohe *et al.*, 1986; Tomita *et al.*, 1987), there has been no study of the expression of PSTI in colorectal cancer, except for the preliminary report (Ogawa *et al.*, 1987). In the present study, we demonstrate that colorectal cancer may also express PSTI, not only by immunohistochemical analysis for detection at the product level but also by *in situ* hybridisation and Northern blot hybridisation for detection at the transcriptional level. In addition, the possible biological and clinical significance of PSTI expression in colorectal cancer is also discussed.

### Materials and methods

### *Tissue preparation*

The tissues were supplied from surgical specimens resected at the 2nd Dept of Surgery, Osaka University Hospital. They were immediately fixed in 10% cold buffered formalin saline for immunohistochemistry and *in situ* hybridisation, then embedded in paraffin.

For Northern blot hybridisation, tumour tissues and normal colonic mucosa, taken from a region distant from the edge of the tumour tissue, were immediately frozen and stored in liquid nitrogen.

Correspondence: M. Higashiyama, Department of Surgery, The Center for Adult Diseases, Osaka, 3 Nakamachi 1-chome, Higashinari-ku, Osaka 537, Japan.
Received 19 April 1990; and in revised form 28 June 1990.

### *Preparation of anti-PSTI antiserum and immunohistochemical study*

Anti-PSTI antiserum used in this study was produced in rabbits as previously described (Kitahara *et al.*, 1980). Its sensitivity and specificity were also confirmed by radioimmunoassay.

Immunohistochemical study was performed according to a modified method of Hsu *et al.* (1981). Briefly, sections were dewaxed in xylene, rehydrated with a series of ethanol solutions, and endogenous peroxidase activity was blocked using 0.3% hydrogen peroxide in methanol for 30 min. After immersion in 3% normal goat serum for 30 min, in order to block non-specific binding, the sections were incubated with primary anti-PSTI antiserum at a dilution of 1:400 overnight at 4°C, and subsequently with biotinylated goat anti-rabbit IgG (Vector) and avidin–biotin peroxidase complex (Vectastain ABC kit, Vector) for 30 min each at room temperature. They were washed in PBS between each incubation step. The peroxidase reaction was applied using 0.02% 3,3′-diaminobenzidine tetrahydrochloride (Sigma; St Louis, MO, USA) in 0.05 M Tris-HCl (pH 7.6) containing 0.01% hydrogen peroxide, producing a brown stain in areas of antibody binding. Negative controls, in which normal rabbit serum was used, were included for each case. As positive controls, PSTI positive specimens from normal pancreas were simultaneously stained.

The degree of PSTI immunoreactivity was semiquantitatively assessed for staining intensity as the percentage of PSTI-positive cancerous cells, more than 30%, (+ +, strongly positive); less than 30%, (+, weakly positive); and 0%, (–, negative).

### *Preparation of PSTI cDNA probe and in situ hybridisation study*

A 378 bp fragment of λTIC-1, which covers a 237-nucleotide amino acid coding region, a 60-nucleotide 5′-non-coding region and a 81-nucleotide 3′-non-coding region, was used as a probe (Tomita *et al.*, 1987). A 585 bp Sau 3A I fragment of pUC 19 vector DNA was used as a negative control probe. Sulphonation of DNA probes for *in situ* hybridisation was performed by treating denatured DNA with a mixture of bisulphite and *o*-methylhydroxylamine (Budowsky *et al.*, 1972).

The procedure used for *in situ* hybridisation is a modification of that which has been reported previously

(Morimoto *et al.*, 1987). Briefly, the sections were dewaxed, rehydrated, and endogenous peroxidase activity was blocked in absolute methanol with 1% hydrogen peroxidase. They were then treated with 0.2 N HCl, heated to 65°C in 2 × SSC (1 × SSC = 0.15 M sodium chloride, 0.015 M tri-sodium citrate, pH 7.0), and digested with proteinase K (5 $\mu g\ ml^{-1}$) for 5 min at 37°C. After acetylation with 0.25% glacial acetic acid in 0.1 M triethanolamine buffer for 20 min, they were prehybridised for 4 h at 42°C. Hybridisation was performed for 12–18 h at 42°C, using a 0.4 ng $\mu l^{-1}$ sulphonated PSTI or pUC19 cDNA probe. The unhybridised probe was thoroughly washed off in 2 × SSC at 37°C for 4–6 h, and finally washed in 0.1 × SSC for 30 min.

The detection of hybrids was performed by the same immunohistochemical method as that for PSTI, using 3% normal horse serum to block non-specific binding, anti-sulphonated DNA antibody (Orgenics, Yavne, Israel) as primary antibody, and biotinylated horse anti-mouse IgG as secondary antibody. The procedure after the avidin–biotin peroxidase complex reaction was the same as mentioned above. Normal human pancreatic tissue was used as positive controls for the detection of PSTI mRNA.

*Northern blot hybridisation*

Total cellular RNA was isolated from the tissues of colorectal cancer and normal colon mucosa, as described by Chirgwin *et al.* (1979). mRNA was purified from total RNA by repeated passage through a type 7 oligo (dT) cellulose column (Pharmacia, Sweden). An aliquot (5 $\mu g$) of mRNA was denatured by heating at 65°C for 15 min in 50% formamide, electrophoresed in a 1% agarose/2.2 M formaldehyde gel as described by Lehrach *et al.* (1977) and then transferred to a nylon filter (Gene Screen Plus, NEN, USA). After prehybridisation at 65°C for several hours, hybridisation with a $^{32}P$-labelled PSTI cDNA probe (1–2 × $10^6$ c.p.m. $ml^{-1}$) was performed at 65°C for 12–18 h. After the filter was washed in 2 × SSC at 65°C for 30 min and then rinsed in 0.1 × SSC at room temperature, it was exposed to X-ray film (Kodak XA-5) for 5 days at − 70°C. As positive control, normal human pancreatic tissue (0.1 $\mu g$ of mRNA) was also applied.

*Statistical analysis*

Statistical comparison was evaluated using the $\chi^2$ test; $P < 0.05$ was considered to be significantly different.

### Results

*Immunohistochemistry on PSTI*

Of the 95 colorectal cancer cases examined, 80 cases (84%) stained positively with varying degrees of immunoreactivity: 18 cases stained weakly, and 62 cases strongly (Figure 1).

The relationship between PSTI expression and histological type is shown in Table I. The incidence of PSTI expression in well or moderately differentiated adenocarcinomas was greater than in the other types.

Table II shows the relationship between PSTI expression and clinicopathological analyses with regard to stage, tumour size, depth of invasion, nodal involvement and liver metastasis. There were no obvious differences in the incidence of PSTI expression among stages, nodal involvement and liver metastasis. However, as tumour size enlarged, PSTI was expressed more frequently and more strongly ($P < 0.01$). Moreover, in cases which showed strong PSTI immunoreactivity, the incidence of PSTI expression gradually increased with the depth of tumour invasion: cases with invasion into the submucosal layer, into the intramuscular layer, within the serosal layer or adventitia, and into the surrounding organs showed incidences of 30, 67, 69 and 78%, respectively. In particular, the incidence of cases invading within the serosal layer was significantly higher than that of cases only invading the submucosal layer ($P < 0.05$).

**Figure 1** Immunohistochemical staining of PSTI in colorectal cancer. Mayer's haematoxylin counter stain. **a**, Well differentiated adenocarcinoma expressing PSTI weakly as indicated by arrows (original magnification × 40). **b**, Well differentiated adenocarcinoma showing strong reactivity with the anti-PSTI antiserum (original magnification × 33).

**Table I** The relationship between PSTI expression and histological type in colorectal cancer

| | PSTI expression | | | |
|---|---|---|---|---|
| *Histology*[a] | − | + | + + | *Incidence (%)* |
| Well–mod | 8 | 16 | 60 | 76/84 (90) |
| Poor | 4 | 1 | 1 | 2/6 (33) |
| Muc | 2 | 1 | 1 | 2/4 (50) |
| Sig | 1 | 0 | 0 | 0/1 (0) |
| Total | 15 | 18 | 62 | 80/95 (84) |

[a]Well–mod: well or moderately differentiated adenocarcinoma. Poor: poorly differentiated adenocarcinoma. Muc: mucinous carcinoma. Sig: signet ring cell carcinoma.

*Detection on PSTI mRNA*

Of the 15 colorectal cancer cases tested, transcripts were detected in 9 cases by *in situ* hybridisation analysis, and in 13 cases by Northern blot hybridisation analysis (Table III).

Using the *in situ* hybridisation method, PSTI mRNAs were detected in colorectal cancerous cells (Figure 2a), while no staining was detected by negative probe (Figure 2b). As positive control, PSTI mRNAs were demonstrated in the acini of the normal pancreas (Figure 2c).

Representative Northern blot hybridisations are also shown in Figure 3. A hybridising band of about 530 nucleotides was detected with varying degrees of density in each lane: 2 (case no. 13), 3 (case no. 5), 4 (case no. 1) and 5 (case no. 6). Lane 6 (case no. 4) was negative for PSTI mRNA. In lane 1, normal pancreatic mRNA was used as a positive control.

**Table II** The relationship between PSTI expression and clinicopathological analyses in colorectal cancer

| | PSTI expression | | | | |
|---|---|---|---|---|---|
| | – | + | + + | Incidence | (%) |
| Stage[a] | | | | | |
| A | 5 (26) | 4 (21) | 10 (53) | 14/19 | (74) |
| B | 1 (4) | 5 (19) | 21 (78) | 26/27 | (96) |
| C | 5 (19) | 3 (12) | 18 (69) | 21/26 | (81) |
| D | 4 (17) | 6 (26) | 13 (57) | 19/23 | (83) |
| Tumour size (cm)[b] | | | | | |
| $T<4$ | 2 (10) | 9 (45) | 9 (45) | 18/20 | (90) |
| $4\leqslant T<8$ | 13 (20) | 8 (12) | 45 (68) | 53/66 | (80) |
| $8\leqslant T$ | 0 (0) | 1 (11) | 8 (89) | 9/9 | (100) |
| Depth of invasion | | | | | |
| Submucosal layer | 3 ((30) | 4 (40) | 3 (30)[c] | 7/10 | (70) |
| Intramuscular layer | 4 (33) | 0 (0) | 8 (67) | 8/12 | (67) |
| Serosal layer or adventitia | 7 (11) | 13 (20) | 44 (69)[c] | 57/64 | (89) |
| Surrounding organs | 1 (11) | 1 (11) | 7 (78) | 8/9 | (89) |
| Nodal involvement | | | | | |
| Negative | 9 (18) | 10 (20) | 31 (62) | 41/50 | (82) |
| Positive | 6 (13) | 8 (18) | 31 (69) | 39/45 | (87) |
| Liver metastasis | | | | | |
| Negative | 12 (16) | 12 (16) | 51 (68) | 63/75 | (84) |
| Positive | 3 (15) | 6 (30) | 11 (55) | 17/20 | (85) |

[a]According to the criteria of Dukes' staging. [b]Significantly different ($P<0.01$). [c]Significantly different ($P<0.05$).

**Table III** Detection of PSTI and its mRNA in colorectal cancer

| | | | | | Size | | PSTI | | |
|---|---|---|---|---|---|---|---|---|---|
| No. | Age | Sex | Hist. | Stage | (cm) | Dep. | IH | ISH | North |
| 01 | 54 | m | well | A | 3.4 | im | + + | + | + |
| 02 | 54 | f | poor | A | 7.5 | im | – | – | + |
| 03 | 60 | m | mod | B | 3.8 | s | + | – | + |
| 04 | 87 | m | mod | B | 4.0 | s | – | – | – |
| 05 | 69 | m | mod | D | 4.2 | s | + + | – | + |
| 06 | 61 | m | mod | B | 4.5 | s | + + | – | + |
| 07 | 55 | f | well | D | 4.5 | s | + + | + | + |
| 08 | 56 | m | well | B | 4.7 | s | + + | + | + |
| 09 | 56 | m | well | C | 5.5 | s | + + | + | + |
| 10 | 50 | m | mod | B | 7.0 | s | + + | + | + |
| 11 | 45 | f | well | B | 7.0 | s | – | – | – |
| 12 | 49 | m | mod | C | 7.0 | s | + + | + | + |
| 13 | 34 | m | well | B | 8.5 | s | + + | + | + |
| 14 | 62 | f | mod | B | 8.5 | s | + + | + | + |
| 15 | 46 | m | mod | B | 11.0 | s | + + | + | + |
| | | | | | | | 12/15 (80%) | 9/15 (60% | 13/15 (87%) |

Hist.: histology, reference to Table I. Stage: Dukes' staging. Dep.: depth of invasion. im: invasion into the intramuscular layer. s: invasion within the serosal layer or adventitia. IH: immunohistochemistry. ISH: *in situ* hybridisation. North: Northern blot hybridisation.

**Figure 2** Moderately differentiated adenocarcinoma strongly showing PSTI mRNA. Mayer's haematoxylin counter stain (original magnification × 100). **a**, *In situ* hybridisation with PSTI cDNA probe. **b**, *In situ* hybridisation with pUC19 probe as a negative control. **c**, Normal human pancreatic tissues showing strong reactivity on *in situ* hybridisation with PSTI cDNA probe as a positive control. Note the absence of staining in islet cells and pancreatic duct cells (I, islet cells; D, pancreatic duct cells, original magnification × 133).

### Discussion

Several studies have now examined the expression of PSTI in various neoplasms (Higashiyama *et al*., 1990; Ogata, 1988; Ogawa *et al*., 1987; Tomita *et al*., 1987; Ueda *et al*., 1989). The presence of PSTI in gynaecological cancer and gastric cancer was immunohistochemically demonstrated using the same anti-PSTI antiserum used in this study (Higashiyama *et al*., 1990; Ueda *et al*., 1989). Using biochemical assays, it was also reported that a pancreatic cancer cell line, CAPAN-1, produces PSTI (Ogata, 1988). When a PSTI cDNA clone has been isolated and sequenced, the expression of PSTI at the gene level can be examined. In fact, it was shown that the transcript of PSTI could be detected in some lung cancer tissues by Northern blot hybridisation analysis (Tomita *et al*., 1987). However, the expression of PSTI in neoplasms has not been simultaneously examined at both the product and the transcriptional level. Besides, by Northern blot hybridisation analysis, although the method is now reliably established, only total admixtures of mRNA, not only from cancerous cells but from stromal cells and/or occasionally non-cancerous epithelial cells in the tissues, can be evaluated. In this respect, the present study appears to be of much value, as the evidence that PSTI is produced and expressed by cancerous cells in colorectal cancerous tissues can be convincingly demonstrated only by simultaneous *in situ* detection of PSTI and PSTI mRNA in addition to Northern blot hybridisation analysis.

While the expression of PSTI was observed in 90% of well or moderately differentiated adenocarcinomas, histologically common types of colorectal cancer, it was observed that the incidence of PSTI expression in histologically uncommon types including poorly differentiated adenocarcinomas, mucinous carcinomas and signet ring cell carcinomas was

**Figure 3** Northern blot hybridisation with PSTI cDNA (lane 1, normal pancreas; lane 2, case no. 13; lane 3, case no. 5; lane 4, case no. 1; lane 5, case no. 6; lane 6, case no. 4).

low, although the number of cases examined was small. In contrast, it has been previously shown that in gastric cancer, poorly differentiated adenocarcinomas or signet ring cell carcinomas contained PSTI at a rather high incidence (Higashiyama *et al.*, 1990). Therefore, the possibility may exist that the expression of PSTI in cancerous cells is highly variable among the organs and/or tissues of primary origin, irrespective of histological differentiation or mucin production.

The biological and clinical significance of PSTI expression in colorectal cancer, although still unknown, is of much interest. Two possibilities may be considered to explain its significance. Firstly, PSTI may act as a proteinase inhibitor against proteolytic enzyme systems, like the trypsin-PSTI system in the pancreas. Tumour invasion is now associated with local production of some proteolytic enzymes by tumorous cells (Tryggvason *et al.*, 1987). Besides, in tumour tissues, such a proteolytic enzyme and its inhibitor occur together, and the latter could be involved in the inhibition of the former enzymatic activity (Kataoka *et al.*, 1989; Strauli *et al.*, 1980; Turpeinen *et al.*, 1988). Tumour-associated trypsin inhibitor, TATI, is a peptide identified in ovarian cancerous extracts, the *N*-terminal amino acid sequence of which is very similar to PSTI (Halila *et al.*, 1987). The ability of TATI to inhibit not only trypsin but also some fibrinolytic enzymes, such as plasmin, urokinase, and tissue-type plasminogen activator, has been recently demonstrated in its physiological concentrations, suggesting that TATI has a possible role in proteolytic control involving these proteinases (Turpeinen *et al.*, 1988). However, there is no evidence that colorectal cancer may produce trypsin, and the reaction between PSTI and these fibrinolytic enzymes is yet to be resolved, although fibrinolytic enzyme activities have recently been observed in colorectal cancerous tissues (Kohga *et al.*, 1985).

Secondly, although conclusive evidence has not yet been obtained, it has been suggested that PSTI itself may be a growth factor-like substance (Niinobu *et al.*, 1986; Ogawa *et al.*, 1987). PSTI can stimulate DNA synthesis in human fibroblasts *in vitro* (Ogawa *et al.*, 1985), and rat cholecystokinin-releasing peptide (CCK-RP), which may be identical to rat PSTI, has been shown to stimulate the growth of 3T3 fibroblasts (Fukuoka *et al.*, 1986). In fact, we recently reported that a strongly positive PSTI-immunoreactivity was detected in the scirrhous type of gastric cancer (Higashiyama *et al.*, 1990). Moreover, McKeehan *et al.* (1986) showed that tumour-associated proteinase inhibitor, ECGF-2a, isolated from human hepatoma cells, the first 25 amino acids of the *N*-terminal sequence of which is identical to PSTI, may possess a function as endothelial cell growth factor. On the other hand, primary amino acid sequence similarities between PSTI and epidermal growth factor (EGF) have been previously demonstrated (Hunt *et al.*, 1974; Sheving, 1983), and the nucleotide sequences of human PSTI mRNA and mouse EGF mRNA have been shown to be highly homologous (Yamamoto *et al.*, 1985). Besides, the binding site for PSTI has been found in various cultured cells, which is distinct from the receptor for EGF (Niinobu *et al.*, 1986). Furthermore, it has been also suggested that PSTI may have an effect on fetal pancreatic development (Fukayama *et al.*, 1986). These findings raise the possibility that PSTI may possess a growth-stimulating function on stromal cells in the neoplastic tissues as well as neoplastic cells. Therefore, it can be speculated that PSTI produced by tumorous cells acts as an 'active' or 'positive' growth-stimulating factor on the development of tumour tissues, rather than in a 'passive' role only like proteinase inhibitor.

Recently, it has been reported that alpha-1-antitrypsin, a trypsin inhibitor, is more frequently expressed in advanced cases of colorectal cancer than in early ones, indicating that it may be a useful biological marker for prognosis of colorectal cancer (Karashima *et al.*, 1990). The role and significance of alpha-1-antitrypsin expression in colorectal cancer has also been unknown, but it is speculated that it may act as an endothelial cell growth factor or a modulator of host—tumour immune response in cancerous tissues (Ades *et al.*, 1982; McKeehan *et al.*, 1986). The present observations that the increased incidence of immunohistochemically strong reactivity for PSTI correlated well with depth of invasion and tumour size may resemble in some respects similar observations for alpha-1-antitrypsin. Thus, like alpha-1-antitrypsin, the expression of PSTI in colorectal cancer may possibly be associated with tumour development. However, this immunohistochemical finding was not confirmed by the transcriptional level of PSTI because of the small number measured.

In conclusion, it was observed that PSTI is commonly produced and expressed in colorectal cancer. Moreover, we have discussed the possible biological and clinical significance of PSTI in colorectal cancerous cells, and the immunohistochemical findings that more advanced cases, with regard to tumour size and depth of invasion, may express more PSTI. These data suggest that PSTI may play a role in colorectal cancer which is closely associated with tumour development.

We thank Dr K. Matsubara at the Institute for Molecular and Cellular Biology, Osaka University, for supplying the probe of PSTI cDNA. This work was supported in part by grants-in-aid for scientific research from the Ministry of Education, Science and Culture of Japan.

### References

ADES, E.W., HINSON, A., CHAPUIS-CELLIER, C. & ARNAUD, P. (1982). Modulation of the immune response by plasma protease inhibitors. I. Alpha-2-macroglobulin and alpha-1-antitrypsin inhibit natural killing and antibody-dependent cell-mediated cytotoxicity. *Scand. J. Immunol.*, **15**, 109.

BOHE, M., BORGSTRÖM, A., LINDSTRÖM, C. & OHLSSON, K. (1986). Pancreatic endoproteases and pancreatic secretory trypsin inhibitor immunoreactivity in human Paneth cells. *J. Clin. Pathol.*, **39**, 786.

BUDOWSKY, E.I., SVERDLOV, E.D. & MONASTYRSKAYA, G.S. (1972). New method of selective and rapid modification of the cytidine residues. *FEBS Letts*, **25**, 201.

CHIRGWIN, L.M., PRYZYBYLA, A.E., MACDONALD, R.J. & RUTTER, W.J. (1979). Isolation of biologically active ribonucleic acid from sources enriched in ribonuclease. *Biochemistry*, **18**, 5294.

FUKAYAMA, M., OGAWA, M., HAYASHI, T. & KOIKE, M. (1986). Development of human pancreas. Immunohistochemical study of fetal pancreatic secretory proteins. *Differentiation*, **31**, 127.

FUKUOKA, S., FUSHIKI, T., KITAGAWA, Y., SUGIMOTO, E. & IWAI, K. (1986). Growth stimulating activity on 3T3 fibroblasts of molecular weight 6500-peptide purified from rat pancreas juice. *Biochem. Biophys. Res. Commun.*, **139**, 545.

HALILA, H., HUHTARA, M.-L., HAGULUND, C., NORDLIG, S. & STENMAN, U.-H. (1987). Tumour associated trypsin inhibitor (TATI) in human ovarian cyst fluid. A comparison with CA125 and CEA. *Br. J. Cancer*, **56**, 153.

HIGASHIYAMA, M., MONDEN, T., OGAWA, M. & 7 others (1990). Immunohistochemical study on pancreatic secretory trypsin inhibitor (PSTI) in gastric carcinomas. *Am. J. Clin. Pathol.*, **93**, 8.

HSU, S.M., RAINE, L. & FRANGER, H. (1981). Use of avidin-biotin-peroxidase complex (ABC) in immunoperoxidase techniques: a comparison between ABC and unlabeled antibody (PAP) procedures. *J. Histochem. Cytochem.*, **29**, 577.

HUNT, L.T., BAKER, W.C. & DAYHOFF, M.O. (1974). Epidermal growth factor: internal dupliction and probable relationship to pancreatic secretory trypsin inhibitor. *Biochem. Biophys. Res. Commun.*, **60**, 1020.

KARASHIMA, S., KATAOKA, H., ITOH, H., MARUYAMA, R. & KOONO, M. (1990). Prognostic significance of alpha-2-antitrypsin in early stage of colorectal carcinomas. *Int. J. Cancer*, **45**, 244.

KATAOKA, H., NABESHIMA, K., KOMADA, N. & KOONO, M. (1989). New human colorectal carcinoma cell lines that secrete proteinase inhibitors *in vitro*. *Virchows Arch. B Cell Pathol.*, **57**, 157.

KAZAL, L.A., SPICER, D.S. & BRAHINSKY, R.A. (1948). Isolation of a crystalline trypsin inhibitor-anticoagulant protein from pancreas. *J. Am. Chem. Soc.*, **70**, 3034.

KITAHARA, T., TAKATSUKA, Y., FUJIMOTO, K. & 3 others (1980). Radioimmunoassay for human pancreatic secretory trypsin inhibitor: measurement of serum pancreatic secretory trypsin inhibitor in normal subjects and subjects with pancreatic diseases. *Clin. Chim. Acta*, **103**, 135.

KOHGA, S., HARVEY, S.R., WEAVER, R.M. & MARKUS, G. (1985). Localization of plasminogen activators in human colon cancer by immunoperoxidase staining. *Cancer Res.*, **45**, 1787.

LEHRACH, H., DIAMOND, D., WOZNEY, J.M. & BOEDTKER, H. (1977). RNA molecular weight determinations by gel electrophoresis under denaturing conditions, a critical reexamination. *Biochemistry*, **16**, 4743.

MCKEEHAN, W.L., SAKAGAMI, Y., HOSHI, H. & MCKEEHAN, K.A. (1986). Two apparent human endothelial cell growth factors from human hepatoma cells are tumor-associated proteinase inhibitors. *J. Biol. Chem.*, **261**, 5378.

MORIMOTO, H., MONDEN, T., SHIMANO, T. & 6 others (1987). Use of sulfonated probes for in situ detection of amylase mRNA in formalin-fixed paraffin sections of human pancreas and submaxillary gland. *Lab. Invest.*, **57**, 737.

NIINOBU, T., OGAWA, M., SHIBATA, T. & 4 others (1986). Specific binding of human pancreatic secretory trypsin inhibitor to various cultured cells. *Res. Commun. Chem. Pathol. Pharmacol.*, **53**, 245.

OGATA, N. (1988). Demonstration of pancreatic secretory trypsin inhibitor in serum-free culture medium conditioned by the human pancreatic carcinoma cell line CAPAN-1. *J. Biol. Chem.*, **263**, 13427.

OGAWA, M., TSUSHIMA, T., OHBA, Y. & 4 others (1985). Stimulation of DNA synthesis in human fibroblasts by human pancreatic secretory trypsin inhibitor. *Res. Commun. Chem. Pathol Pharmacol.*, **50**, 155.

OGAWA, M., MATSURA, N., HIGASHIYAMA, K. & MORI, T. (1987). Expression of pancreatic secretory trypsin inhibitor in various cancer cells. *Res. Commun. Chem. Pathol. Pharmacol.*, **55**, 137.

SCHEVING, L.A. (1983). Primary amino acid sequence similarity between human epidermal growth factor-urogastrone, human pancreatic secretory trypsin inhibitor, and members of porcine secretion family. *Arch. Biochem. Biophys.*, **226**, 411.

STRAULI, P., BARRETT, A.J. & BAICI, A. (1980). *Proteinases and Tumour Invasion, vol. 6*. Raven Press: New York.

TOMITA, N., HORII, A., YAMAMOTO, T. & 3 others (1987). Expression of pancreatic secretory trypsin inhibitor gene in neoplastic tissues. *FEBS Letts*, **225**, 113.

TRYGGVASON, K., HOYHTYA, M. & SALO, T. (1987). Proteolytic degradation of extracellular matrix in tumor invasion. *Biochim. Biophys. Acta*, **907**, 191.

TURPEINEN, U., KOIVUNEN, E. & STENMAN, U.-H. (1988). Reaction of a tumour-associated trypsin inhibitor with serine proteinases associated with coagulation and tumour invasion. *Biochem. J.*, **254**, 911.

UEDA, G., SHIMIZU, C., TANAKA, Y. & 4 others (1989). Immunohistochemical demonstration of pancreatic secretory trypsin inhibitor in gynecological tumors. *Gynecol. Oncol.*, **32**, 37.

YAMAMOTO, T., NAKAMURA, Y., NISHIDE, T. & 4 others (1985). Molecular cloning and nucleotide sequence of human pancreatic secretory trypsin inhibitor (PSTI) cDNA. *Biochem. Biophys. Res. Commun.*, **132**, 605.